# The Clinical and Radiological Spectrum of Hippocampal Pathology in Amyotrophic Lateral Sclerosis

**DOI:** 10.3389/fneur.2018.00523

**Published:** 2018-07-03

**Authors:** Foteini Christidi, Efstratios Karavasilis, Georgios Velonakis, Panagiotis Ferentinos, Michail Rentzos, Nikolaos Kelekis, Ioannis Evdokimidis, Peter Bede

**Affiliations:** ^1^First Department of Neurology, Aeginition Hospital National and Kapodistrian University of Athens, Athens, Greece; ^2^Second Department of Radiology, General University Hospital “Attikon”, National and Kapodistrian University of Athens, Athens, Greece; ^3^Second Department of Psychiatry, General University Hospital “Attikon”, National and Kapodistrian University of Athens, Athens, Greece; ^4^Computational Neuroimaging Group, Academic Unit of Neurology, Trinity College Dublin, Dublin, Ireland

**Keywords:** hippocampus, amyotrophic lateral sclerosis, neuropathology, neuroimaging, cognition

## Abstract

Hippocampal pathology in Amyotrophic Lateral Sclerosis (ALS) remains surprisingly under recognized despite compelling evidence from neuropsychology, neuroimaging and neuropathology studies. Hippocampal dysfunction contributes significantly to the clinical heterogeneity of ALS and requires structure-specific cognitive and neuroimaging tools for accurate *in vivo* evaluation. Recent imaging studies have generated unprecedented insights into the presymptomatic and longitudinal processes affecting this structure and have contributed to the characterisation of both focal and network-level changes. Emerging neuropsychology data suggest that memory deficits in ALS may be independent from executive dysfunction. In the era of precision medicine, where the development of individualized care strategies and patient stratification for clinical trials are key priorities, the comprehensive review of hippocampal dysfunction in ALS is particularly timely.

## Introduction

Amyotrophic lateral sclerosis (ALS) is relentlessly progressive neurodegenerative condition with considerable clinical heterogeneity (1). One of the key clinical dimensions of disease heterogeneity in ALS is the varying severity and profile of cognitive impairment. The quality of life implications of cognitive impairment in ALS and its impact on caregiver burden (2), compliance with assistive devices (3) and survival (4) are now universally recognized. The discovery of hexanucleotide expansions in *C9orf72* in 2011 (5) has given fresh momentum to neuropsychology research in ALS by confirming shared etiological factors between frontotemporal dementia (FTD) and ALS. The momentous conceptual advances in the neuropsychology of ALS have taken place in a remarkably short period of time, from sporadic observations, through the development of diagnostic criteria (6), to robust family aggregation (7) and genetic studies, to the development of disease-specific screening instruments (8, 9). The current consensus criteria (6) distinguish ALS with cognitive impairment; ALS with behavioral impairment; ALS with cognitive and behavioral impairment; ALS-FTD; ALS-dementia (non-FTD, i.e., Alzheimer dementia (AD), vascular dementia,mixed dementia). One of the most exciting aspects of ALS neuropsychology studies is their localization potential to specific anatomical circuits and that their observations are widely corroborated by neuropathology (10–12) and neuroimaging studies (13). Memory deficits in ALS have traditionally been regarded as atypical and considered suggestive of coexisting AD-type pathology. The recognition that memory deficits are part of the spectrum of ALS-associated cognitive impairment is relatively recent.

## Memory impairment in ALS

Early neuropsychology studies of ALS have predominantly examined frontal lobe-mediated neuropsychological domains, and highlighted executive dysfunction, impaired phonemic fluency, poor set shifting, reduced cognitive flexibility, impaired response inhibition, planning deficits, problem-solving difficulties, selective attention, and impaired social cognition (14). More recently, the spectrum of memory impairment has been specifically evaluated, including encoding and retrieval functions (primary memory system) (15, 16) and storage/consolidation domains (secondary memory system) (17). Furthermore, population-based studies identified cognitive phenotypes without executive impairment (18, 19). The description of episodic memory deficits without coexisting executive dysfunction in ALS drew attention to temporal lobe network dysfunction which has been elegantly corroborated by a series of neuropathology and neuroimaging studies (20).

## Anatomical overview

**Figure 1:**
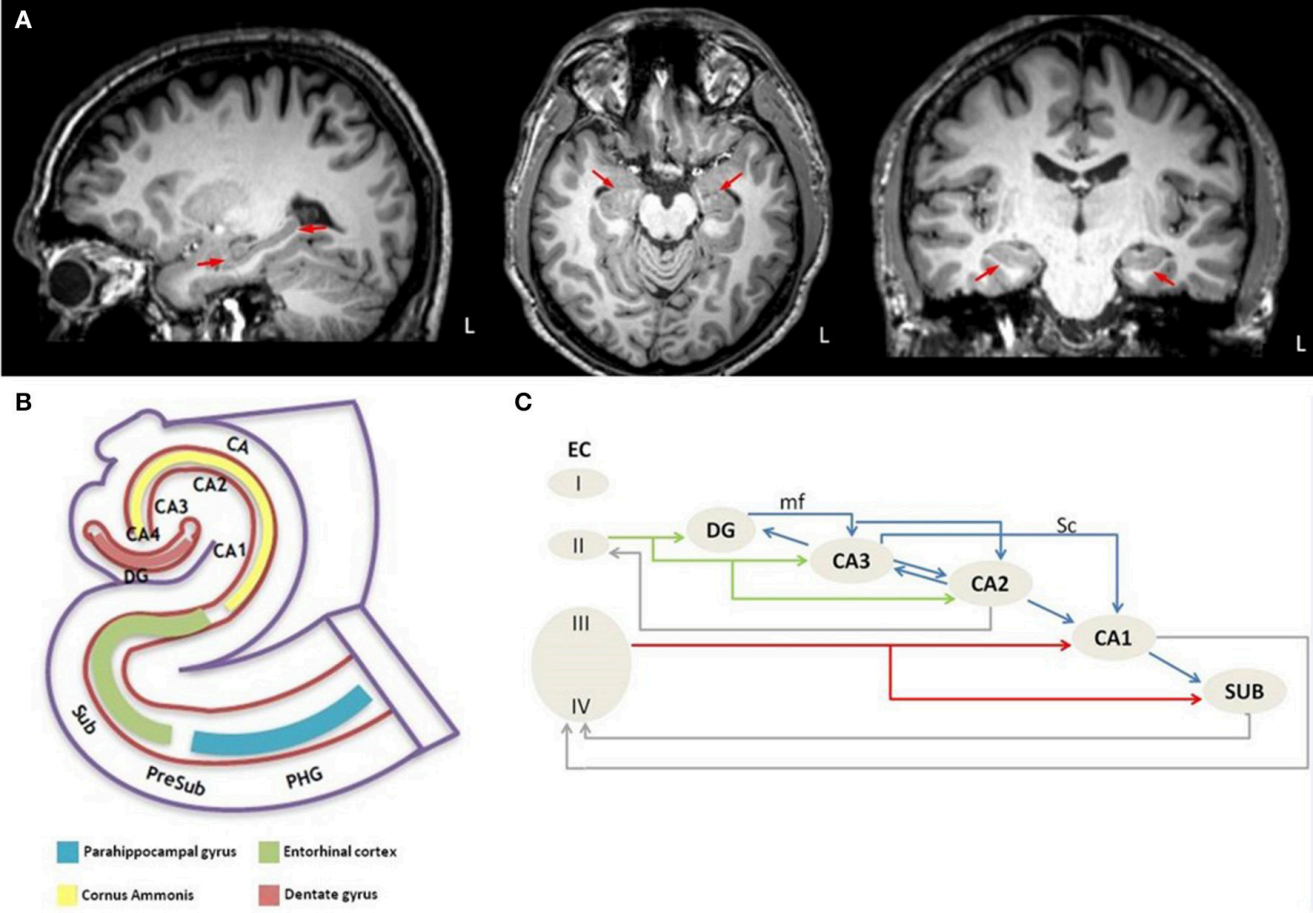
Anatomical depiction of hippocampus on sagittal, axial and coronal plane of high resolution T1 image from a healthy control subject **(A)** and schematic representation of **(B)** the anatomy of the hippocampus-entorhinal cortex-parahippocampal gyrus system and **(C)** the intrahippocampal connections. L, left hemisphere; PHG, parahippocampal gyrus; PreSub, Presubiculum; Sub, Subiculum; CA, Cornu Ammonis; CA1-CA4, Cornu Ammonis subfields; DG, Dentate gyrus; EC, Entorhinal cortex; I-IV, Layer I-IV; mf, mossy fibers; Sc, Schaffer collaterals.

The hippocampus (Figure 1A) is a bilaminar structure and consists of the cornu ammonis (CA) and the dentate gyrus (DG). Based on its cytoarchitecture and projections, the CA is further divided into four histological subfields, named CA1-CA4 by Lorente de No in his seminal paper (21). The dentate gyrus is a narrow, dorsally concave structure which envelops CA4. The cornu ammonis, the dentate gyrus, and the subiculum together form the “hippocampal formation” (Figure 1B). The subiculum is divided into the following segments: the prosubiculum, the subiculum proper, the presubiculum, and the parasubiculum.

Each segment of the hippocampal formation receives afferentation from its neighboring regions but these connections are not all bidirectional (22). For example, the “trisynaptic circuit” (23) is a unidirectional network, which arises from layer II of the entorhinal cortex, its axons perforate the subiculum, and form the “perforant pathway” (PP). Duvernoy (24) coined the term “polysynaptic pathway” for the intrinsic hippocampal circuitry, which refers to a circuit of at least four synapses that connect the entorhinal cortex, the dentate gyrus, the CA subfields, and the subiculum. A direct intrahippocampal pathway has also been identified, which originates from layer III of the entorhinal cortex and projects directly to the CA1 but not through the PP (25). The perforant pathway (Figure 1C) arises from layer II-III neurons of the lateral and medial entorhinal cortex (26), which is also the origin of the polysynaptic pathway (27). The PP perforates the subiculum to reach the dentate gyrus and the hippocampus proper, but minor projections also originate from the presubiculum and parasubiculum (28). The majority of the PP fibers reach the stratum moleculare of the dentate gyrus through the vestigial hippocampal sulcus (24). The PP contributes to the “Papez circuit” (26, 29, 30) which is relayed through the following structures; entorhinal cortex → dentate gyrus → hippocampus → hypothalamus → thalamus → cingulate cortex → presubiculum → entorhinal cortex. In addition to the intrinsic hippocampal circuitry, there are numerous extrinsic hippocampal projections to subcortical and cortical regions (31). The main input to the hippocampus enters via the entorhinal area (31).

## Insights from neuropathology

Neuropathological changes have been consistently reported in the hippocampus in ALS (Table 1). Early reports highlighted ubiquitin-positive intraneuronal inclusions (32–35) in medial temporal structures, neuronal loss in the medial cortex of the temporal tip (36, 69) and focal depletion of pyramidal neurons in the pes hippocampi in both patients with and without dementia (33, 36, 69). A specific focus of interest in histopathological studies is the PP zone, which has been comprehensively studied in most neurodegenerative conditions, especially in AD. While AD is characterized by the extraneuronal deposits of the amyloid β-protein (Aβ) and the intraneuronal tauopathy (70), ALS is primarily associated with TAR DNA-binding protein 43 (TDP-43) deposits (71). ALS patients with and without dementia (37, 38) show neuropathological changes in the dentate gyrus and the outer lamina of the molecular layer where the PP terminals are distributed (26, 30, 72). In ALS, the molecular layer of the dentate gyrus is primarily affected, a pattern which is distinctly different from AD. The inner molecular layer, which is innervated by the CA4 (73), is the least affected layer in ALS (38). The intermediate layer, which receives projections from layer II of the medial entorhinal cortex, is affected (38), but the outer layer, is the most affected region in ALS (38). Despite considerable mesial temporal lobe involvement in both ALS and AD, the distribution of disease-specific inclusions is strikingly different. Neurofibrillary tangles in AD are mostly found in the entorhinal cortex and are positive for tau, whereas the main proteinopathy of ALS is TDP-43 and mostly affects the transentorhinal cortex (38).

**Table 1:** Research studies with hippocampal-related neuropathological, neuroimaging, or neuropsychological findings in ALS included in the present review.

**References**	**Authors (Date)**	**Sample size**	**Diagnostic criteria**	**Genetic Status**	**Dementia**	**Cognitive status**
**NEUROPATHOLOGICAL STUDIES**						
(32)	Wightman et al., 1992	33 ALS	N/A	N/A	Included	19 PtwoCI; 14 PtwCI-D^*^
(33)	Okamoto et al., 1991	27 ALS/50 HC	N/A	N/A	1 PtwD	N/A (1PtwD)^*^
(34)	Okamoto et al., 1992	10 MND	N/A	N/A	10 PtwD	Dementia^*^
(35)	Okamoto et al., 1996	2 ALS	N/A	N/A	N/A	Mental changes^*^
(36)	Nakano et al., 1993	54 ALS/35 non ALS	N/A	N/A	10 PtwD	44 PtwoD; 10 PtwD
(37)	Takeda et al., 2007	12 ALS	N/A	N/A	12 PtwD	Demented
(38)	Takeda et al., 2009	14 ALS	N/A	N/A	9 PtwD	9 PtwD^*^ (6 PtwMI)
(39)	Brettschneider et al., 2012	102 ALS	El Escorial-R	N/A	12 PtwD	88 PtwoD; 12 PtwD; 2 unknown
(12)	Brettschneider et al., 2013	76 ALS	El Escorial-R	11 C9orf72(+)	5 PtwD	71 PtwoD^*^ 5 PtwD^*^
**NEUROIMAGING STUDIES: STRUCTURAL GM**						
(40)	Bede et al., 2013	39 ALS/44 HC	El Escorial	9 C9orf72(+)	N/A	Cognitive exam; Unspecified cognitive groups
(41)	Abdulla et al., 2014	58 ALS/29 HC	El Escorial-R	3 C9orf72(+)	N/A	Cognitive exam; Unspecified cognitive groups
(42)	Machts et al., 2015	67 ALS/ 39 HC	El Escorial-R	C9orf72(-)	7 PtwD	Cognitive exam; 42 PtwoCI; 18 PtwCI; 7 PtwFTD
(43)	Westeneng et al., 2015	112 ALS/60 HC	El Escorial-R	7 C9orf72(+)	N/A	N/A
(44)	Sage et al., 2007	28 ALS/26 HC	El Escorial	N/A	PtwoD	No behavioral or cognitive changes; Unspecified cognitive exam
(45)	Sage et al., 2009	28 ALS/26 HC	El Escorial	N/A	PtwoD	No behavioral or cognitive changes; Unspecified cognitive exam
**NEUROIMAGING STUDIES: STRUCTURAL WM**						
(46)	Barbagallo et al., 2014	24 ALS/22 HC	El Escorial-R	N/A	N/A	13 Pt cognitively examined; Unspecified cognitive groups
(47)	Thivard et al., 2007	15 ALS/25 HC	El Escorial-R	N/A	PtwoD	N/A
(48)	Prell et al., 2013	17 ALS/17 HC	El Escorial-R	N/A	PtwoD	No significant frontal or cognitive dysfunction; Unspecified cognitive exam
(49)	Keil et al., 2012	24 ALS/24 HC	El Escorial-R	N/A	PtwoD	No cognitive exam
(50)	Kassubek et al., 2014	111 ALS/74 HC	El Escorial-R	N/A	N/A	N/A
(51)	Christidi et al., 2017	42 ALS/25 HC	El Escorial-R	N/A	PtwoD	Cognitive exam; Memory impairment based on normative data; Unspecified cognitive groups
(52)	Steinbach et al., 2015	16 ALS/16HC	El Escorial-R	N/A	16 PtwD	Cognitive exam; Cognitive categories based on Phukan criteria^*^^#^
**NEUROIMAGING STUDIES: TASK fMRI**						
(53)	Stoppel et al., 2014	14 ALS/14 HC	El Escorial-R	N/A	PtwoD	Cognitive exam; Memory impairment based on normative data; Cognitive categories based on Phukan criteria^*^^#^
**NEUROIMAGING STUDIES: RESTING-STATE fMRI**						
(54)	Agosta et al., 2011	26 ALS/15 HC	El Escorial-R	N/A	PtwoD	N/A
(55)	Zhu et al., 2015	22 ALS/22 HC	El Escorial-R	N/A	PtwoD	Cognitive exam; Unspecified cognitive groups
(56)	Heimrath et al., 2014	9 ALS/11 HC	El Escorial-R	N/A	PtwoD	Cognitive exam; Unspecified cognitive groups
(57)	Loewe et al., 2017	64 ALS/38 HC	El Escorial-R	N/A	PtwoD	Cognitive exam; Specified cognitive groups^*^^#^
**NEUROPSYCHOLOGICAL STUDIES**						
(58)	Abrahams et al., 1997	12 ALS/25 HC	N/A	N/A	N/A	Cognitive exam; Unspecified cognitive groups
(59)	Chari et al., 1996	50 MND/27 HC/23 NeuroC	El Escorial	N/A	PtwoD	Cognitive exam; Unspecified cognitive groups
(60)	Frank et al., 1997	74 ALS/56 HC	N/A	N/A	N/A	Cognitive exam; Unspecified cognitive groups
(61)	Hanagasi et al., 2002	20 ALS/13 HC	El Escorial	N/A	PtwoD	Cognitive exam; Unspecified cognitive groups
(62)	Iwasaki et al., 1990	18 ALS/15 HC	N/A	N/A	PtwoD	Cognitive exam; Unspecified cognitive groups
(63)	Ludolph et al., 1992	17 ALS/12 HC	N/A	N/A	PtwoD	Cognitive exam; Unspecified cognitive groups
(64)	Massman et al., 1996	146 ALS	El Escorial	N/A	N/A	Cognitive exam; Cognitive impairment based on normative data; Unspecified cognitive groups
(65)	Mantovan et al., 2003	20 ALS/20 HC	El Escorial	N/A	PtwoD	Cognitive exam; Unspecified cognitive groups
(66)	Christidi et al., 2012	22 ALS/22 HC	El Escorial-R	N/A	PtwoD	Cognitive exam; Unspecified cognitive groups
(67)	Machts et al., 2014	40 ALS/39 aMCI/40 HC	El Escorial-R	N/A	3 PtwD	Cognitive exam; Unspecified cognitive groups
(68)	Burke et al., 2017	203 ALS/117 HC	El Escorial-R	C9orf72(–)	30 PtwD	Cognitive exam; 117 PtwoCI; 56 PtwCI; 30 PtwD

ALS, amyotrophic lateral sclerosis; HC, healthy control; MND, motor neuron disease; N/A, non-available; PtwoCI, patients without cognitive impairment; PtwCI-D, patients with cognitive impairment-dementia; PtwD, patients with dementia; PtwoD, patients without dementia; PtwMI, patients with memory impairment; PtwCI, patients with cognitive impairment; PtwFTD, patients with frontotemporal dementia;

* *unspecified cognitive status*;

# *no comparison between cognitive groups; El Escorial-R, El Escorial revised criteria; C9orf72(+), C9orf72 positive status; GM, gray matter; WM, white matter*.

It is now widely recognized that phosphorylated TDP-43 (pTDP-43) aggregates are the hallmark pathology of sporadic ALS (39, 74, 75). Based on post mortem observations, a sequential staging system of pTDP-43 pathology has been proposed, using stage-defining involvement of specific cortical and subcortical regions (12). According to this four-stage model of disease propagation, the PP is predominantly affected in stage IV. A three-stage model has also been suggested for PP degeneration (38) where stage I is the “inclusion stage” defined by TDP-43-positive cytoplasmatic inclusions appearing in the granular cells of the dentate gyrus, stage II is the “early perforant stage” where gliosis and neuronal loss of the transentorhinal cortex are observed, and stage III is the “advanced perforant stage” defined by the degeneration of the molecular layer of the dentate gyrus and neuronal loss in the transentorhinal cortex (38). The chronological stages of hippocampal pathology are closely linked to its structural anatomy, confirming that disease propagation occurs along connectivity patterns (76). The TDP-43 stages of ALS are in line with the notion that gray matter (GM) regions become sequentially involved via the WM pathways that connect them (77–79).

## The contribution of neuroimaging

Neuroimaging studies have already contributed meaningful structural, metabolic and functional insights in ALS (80, 81) and recent technological advances in imaging techniques offer unprecedented opportunities to characterize hippocampal changes *in vivo*. Following sporadic reports of hippocampal degeneration (82, 83, 84, 85) in whole-brain exploratory studies, recent studies have specifically focused on the evaluation of this structure (43) (Table 1). Emerging imaging methods not only highlight hippocampal volume reductions, but have the potential to characterize specific sub-regions (78), shape changes (42), density alterations (20), progressive longitudinal changes (43), altered connectivity profiles, and functional changes (40, 46, 47).

### Structural neuroimaging

Computational neuroimaging techniques have consistently captured hippocampal GM changes which was initially thought to be more significant in ALS patients carrying the *C9orf72* hexanucleotide repeats (40), but later studies showed similarly extensive hippocampal degeneration in *C9orf72* negative ALS-FTD patients (78). Interestingly, unilateral hippocampal changes were not only captured in patients with cognitive impairment (42), but also in cognitively intact cohorts (41). Shape and density analyses of the hippocampus in ALS highlighted phenotype-specific patterns of hippocampal degeneration (42). A longitudinal study of hippocampus, which included a small (~6%) number of *C9orf72* positive patients, identified baseline changes in the left presubiculum, and progressive CA2/3, CA4 and the left presubiculum involvement at follow-up (43).

While diffusion-weighted imaging (DWI) is primarily used to study white matter (WM) structures, there is increasing evidence that it may provide useful information on aspects of GM integrity (86). Evaluation of diffusion tensor imaging (DTI) metrics have consistently shown low fractional anisotropy (44, 49) and increased mean diffusivity in both hippocampal (44–47) and parahippocampal regions (48).

DTI has been initially used to characterize medial temporal lobe WM regions and later to assess limbic circuit integrity (i.e., fornix; uncinate fasciculus) (87,–89). One of the most unique applications of hippocampal DTI in ALS however is the ability to reconstruct and evaluate of the PP. (50, 51). Based on *in vivo* assessments, these studies have not only confirmed previous neuropathological observations but also revealed structure-specific clinical correlations (51). The use of DWI-based PP imaging (90) has contributed to our understanding of impaired memory processing in a range of conditions from mild cognitive impairment, through AD, to traumatic brain injury (91,–94). PP imaging is therefore a relatively well-established approach which has only recently been applied to ALS. A longitudinal tractography study of ALS (52) found increased connectivity between the visual cortex and medial temporal lobe regions which increased further at 3-month follow-up. Increased connectivity over time in ALS is not an isolated finding (95) and is often interpreted as a compensatory mechanism.

### Functional neuroimaging

There are relatively few paradigm-based functional magnetic resonance imaging (fMRI) studies specifically evaluating hippocampal function, but a longitudinal fMRI study identified increased novelty-evoked hippocampal activity over time (53). Resting-state studies have consistently captured increased connectivity between the left sensorimotor cortex and contralateral cortical regions including the parahippocampal gyrus (54). Additionally, increased low-frequency amplitudes have been observed in the right parahippocampal cortex (55). Increased functional connectivity was also identified between parahippocampal components of the default-mode network (56). In a relatively large sample of ALS patients with only minor cognitive changes, (57) decreased functional connectivity was identified between temporal lobe structures, including hippocampal and parahippocampal regions. This was thought to represent early metabolic disturbances before cell-loss occurs but highlight the fact that increased and decreased connectivity is both reported in fMRI studies of ALS.

## Insights from neuropsychology

Contrary to the consensus around executive dysfunction in ALS (96–99), there are strikingly inconsistent reports about the incidence of memory impairment in ALS (Table 1). Intact memory function, mild dysfunction, executive function mediated memory impairment, and moderate memory deficits have all been reported (58–65, 97). The primary substrate of amnestic deficits is still under investigation. Most studies agree that the primary deficit is in encoding-retrieval (65) which is often linked to frontal dysfunction, attention, and other executive-based processes (65–68). However, recognition deficits and memory consolidation difficulties are likely to be just as important (66). Compelling evidence also exist for pure episodic memory dysfunction based on impaired picture recall, word list-learning, pair associations, and story-recall. These observations would suggest that memory impairment in non-demented ALS patients cannot be exclusively attributed to executive dysfunction (100–102).

In a combined neuroimaging-neuropsychology study, abnormal immediate and delayed recall scores were identified in 23% of non-demented ALS patients (102). While the ALS cohort of this study did not exhibit reduced hippocampal volumes in comparison to healthy controls, their memory performance correlated with hippocampal volumes. These findings are echoed by other studies which rely on volumetric analyses and verbal list-learning test and report significant correlations between the hippocampal volumes and verbal memory indices such as total learning, delayed recall, and recognition (41).

While direct clinico-radiological correlations are often regarded as contentious (103), a positive association has been reported between verbal memory indices and hippocampal volumes in several ALS subgroups, including ALSci and ALS-FTD (42). DTI studies have consistently revealed correlations between memory performance and memory-associated WM tracts such as the fornix (88), the uncinate fasciculus (87, 88), and the hippocampal PP (51). Emerging reports of similar episodic memory performance in ALS and amnestic mild cognitive impairment patients (67) corroborates neuropathological findings of comparable PP changes (37, 38).

### Testing recommendations

Traditionally, the assessment of episodic memory includes tests for immediate and delayed recall, and performance evaluated from a learning, retention and recognition perspective. More recently, distinct memory processes are specifically assessed, such as encoding, consolidation, and retrieval. (104–106) List-learning tests (e.g., California Verbal Learning Test; Rey Auditory Verbal Learning Test; Hopkins Verbal Learning Test etc.) are particularly useful to assess hippocampus-mediated verbal memory dysfunction in ALS. These tests enable the clinician to evaluate immediate recall, delayed recall, and recognition and can be readily interpreted in terms of encoding, consolidation, and retrieval performance (66). Story-recall tests, such as the Wechsler-Memory Scale, are also sensitive to detect episodic memory impairment and ideally, both list-learning and story-recall should be performed to comprehensively evaluate episodic memory in ALS. The accurate assessment of visual episodic memory is often confounded by motor disability in in ALS or by coexisting executive dysfunction which may affect the organization and encoding of complex figures (e.g., Rey-Osterreith Complex Figure Test). The limitations of short, non-ALS, cognitive screening tools such as MMSE; ACE; MoCA are widely recognized in the ALS research community, as these tests have been developed for other neurodegenerative conditions. The administration of ALS specific screening tools (ECAS, ALS-CBS) should be followed by specialist neuropsychological evaluation if memory impairment is identified or reported by the patient or caregiver.

## Discussion

The synthesis of insights from neuropathology, neuroimaging and neuropsychology enables the systematic discussion of structural and functional aspects of hippocampal degeneration in ALS and helps to integrate focal pathology into a network perspective.

While hippocampal pathology used to be primarily evaluated in ALS patients with comorbid dementia (34, 37, 38), recent studies have increasingly focused on non-demented patient cohorts (12, 32, 69, 71). With the increased recognition of neuropsychological deficits beyond executive dysfunction, imaging studies of ALS have gradually started to evaluate mesial temporal lobe structures and memory domains have now been incorporated in ALS-specific cognitive screening tools (8). The targeted evaluation of memory function and reliance on more sophisticated indices of episodic memory (65–68) not only help to characterize the heterogeneity of cognitive profiles but also confirm that pure episodic memory dysfunction is not uncommon in ALS and can be detected in the absence of FTD.

Despite the momentous advances in characterizing hippocampal degeneration in ALS, considerable shortcomings and inconsistencies can be identified. The commonest problem is sample size limitations followed by the inclusion of poorly characterized patients. The comprehensive neuropsychological assessment of patients is paramount and administering screening tests alone is not sufficient. Reliance on non-ALS specific batteries, such as Addenbrooke's Cognitive Examination, Mini-Mental State Examination, Montreal Cognitive Assessment, is not sufficient to characterize ALS-associated cognitive change. A common shortcoming of ALS neuropsychology papers is overlooking the confounding effect of medications which affect cognitive performance. Anticholinergics commonly used for sialorrhea, tricyclic antidepressants, opiates, benzodiazepines are all widely used in ALS and have a significant impact on attention, registration, and recall. Other disease-specific confounding factors such as hypoxia, hypercapnia, physical discomfort, fatigue, apathy, low mood, depression also need careful consideration. Despite established consensus criteria (6) different batteries are used in different centers to test memory. There is a paucity of reports where caregivers or family members are interviewed about the sort of memory impairment they may have observed. A few targeted questions if the patient gets lost in familiar places, misplaces items, forgets names, or dates etc. may be worth asking from the caregivers. Given the strikingly quick progression rates observed in ALS compared to other neurodegenerative conditions, resource allocation, care planning, assessment of capacity may be important at an early stage of the disease. ALS patients have to make a number of important financial, personal, and end-of-life decisions which may or may not be affected by memory impairment.

The practice of excluding patients with dementia in neuroimaging studies (47, 44, 49, 55) to evaluate clinically homogenous samples may also be counterintuitive. More recent imaging papers include comprehensive cognitive testing (55–57) which aids the interpretation of extra-motor changes (107). The lack of cognitive profiling of the healthy controls in many neuroimaging studies also precludes robust statistics as only the patient group is then used for correlative analyses. Often, reference normative neuropsychology data are used for the interpretation of patient's memory performance, data which is independent from the given study and originate from volunteers who have not been scanned as part of the given study. The patients' neuroimaging data on the other hand are contrasted to scans of controls who had no detailed neuropsychological evaluation. This unfortunately is a common study design, which essentially uses a different imaging and neuropsychology control group. Another common shortcoming of ALS neuroimaging studies is the lack of adjustment for education, which may impact on both structural and functional imaging data (80). A binary, comparative study design of patients versus controls and the contrasting of two clinically or genetically defined cohorts is not entirely satisfactory either. The inclusion of mimic cohorts, or a “disease-control” group with an alternative neurodegenerative condition such as MCI, AD, or Parkinson disease would also be desirable. The selection bias of relatively well patients who are able to lie flat in the scanner and able to make the journey to a radiology department is seldom acknowledged. It is conceivable that progressive hippocampal changes occur as the disease progresses, but these patients are no longer able to partake in imaging studies. Clinical trial designs are not only hampered by late recruitment of clinically heterogeneous cohorts, but they overwhelmingly rely on motor, respiratory, nutritional markers (108–110). Patient stratification based on cognitive performance prior to inclusion and monitoring performance during the trial seems essential, especially given the survival implications of cognitive impairment (3, 4, 111).

Despite initial enthusiasm that hexanucleotide repeats account for most of the ALS-FTD cohort (112, 113), it has quickly become apparent that *C9orf72* hexanucleotide repeats only explain a minority of ALS-FTD cases (114). Emerging studies confirm that a subgroup of *C9orf72* negative patients may show neuroanatomical alterations similar to the ones observed in patients carrying the hexanucleotide expansion. Furthermore, temporal lobe changes have been captured in asymptomatic hexanucleotide carriers, who also exhibited subcortical gray matter degeneration prior to symptom onset (115).

Existing multimodal studies which combine neuroimaging and neurocognitive measures either support a close association between anatomical changes and memory performance or highlight a relative dissociation between the two methods. This inconsistency is epitomized by reports of absent neuroimaging changes in patients with established memory deficits and the detection of significant hippocampal changes in patients with mild memory impairment (41, 42, 102). Based on the shortcomings of existing hippocampal studies in ALS, future studies should include large sample sizes, disease-controls, longitudinal designs, paradigm-based fMRI, comprehensive neuropsychological profiling, “disease-controls,” anatomical corrections for education, and genetic screening for mutations implicated in ALS, FTD, and AD. Furthermore, reliance on high directional diffusion models such as neurite orientation dispersion and density imaging (NODDI), high angular resolution diffusion imaging (HARDI), or Q-ball imaging may be desirable to characterize early WM alterations in parahippocampal regions. Finally, combined imaging and post-mortem studies may provide a validation of the *in vivo* findings.

In conclusion, hippocampal pathology is a clinically and academically relevant field of ALS research which has gained unprecedented momentum in recent years and is likely to contribute important further insights in the coming years.

## Author contributions

The paper was drafted by FC, EK, and PB and has been reviewed for intellectual content by GV, PF, MR, NK, and IE.

### Conflict of interest statement

The authors declare that the research was conducted in the absence of any commercial or financial relationships that could be construed as a potential conflict of interest.

## Abbreviations

ALS: amyotrophic lateral sclerosis
ALSFRS-r: revised ALS functional rating scale revised
ALSnci: ALS with no cognitive impairment
aMCI: amnestic mild cognitive impairment
C9orf72 HRT: chromosome 9 open reading frame 72 hexanucleotide repeats
CA: cornu ammonis
DG: dentate gyrus
DTI: diffusion tensor imaging
DWI: diffusion weighted imaging
ECAS: Edinburgh cognitive and behavioral ALS screen
FTD: frontotemporal dementia
GM: gray matter
HARDI: high angular resolution diffusion imaging
HC: healthy control
MND: motor neuron disease
NeuroC: neurological controls
NODDI: neurite orientation dispersion and density imaging
PP: perforant pathway
PtwoCI: patients without cognitive impairment
PtwCI: patients with cognitive impairment
PtwoD: patients without dementia
PtwD: patients with dementia
TBSS: tract-based spatial statistics
VBM: voxel-based morphometry
WM: white matter
MRS: magnetic resonance spectroscopy.
